# Rearward-Facing Infant Child Restraint Systems with Support Legs in Frontal and Frontal-Oblique Impacts

**DOI:** 10.3390/ijerph182010799

**Published:** 2021-10-14

**Authors:** Declan A. Patton, Aditya N. Belwadi, Jalaj Maheshwari, Kristy B. Arbogast

**Affiliations:** 1 Center for Injury Research and Prevention, Children’s Hospital of Philadelphia, Philadelphia, PA 19146, USA; adityabn@gmail.com (A.N.B.); maheshwarj@chop.edu (J.M.); arbogast@chop.edu (K.B.A.); 2 Perelman School of Medicine, University of Pennsylvania, Philadelphia, PA 19104, USA

**Keywords:** anthropomorphic test device, child restraint, motor vehicle crash, sled testing

## Abstract

Previous studies of support legs in rearward-facing infant CRS models have focused on frontal impacts and have found that the presence of a support leg is associated with a reduction in head injury metrics. However, real-world crashes often involve an oblique principal direction of force. The current study used sled tests to evaluate the effectiveness of support legs in rearward-facing infant CRS models for frontal and frontal-oblique impacts with and without a simulated front row seatback. Frontal and frontal-oblique impact sled tests were conducted using the simulated Consumer Reports test method with and without the blocker plate, which was developed to represent a front row seatback. The Q1.5 anthropomorphic test device (ATD) was seated in rearward-facing infant CRS models, which were tested with and without support legs. The presence of a support leg was associated with significant reductions of head injury metrics below injury tolerance limits for all tests, which supports the findings of previous studies. The presence of a support leg was also associated with significant reductions of peak neck tensile force. The presence of the blocker plate resulted in greater head injury metrics compared to tests without the blocker plate, but the result was non-significant. However, the fidelity of the interaction between the CRS and blocker plate as an adequate representation of the interaction that would occur in a real vehicle is not well understood. The findings from the current study continue to support the benefit of support legs in managing the energy of impact for a child in a rearward-facing CRS.

## 1. Introduction

Correct usage of a child restraint system (CRS) is associated with a substantial reduction of injury and mortality risks in motor vehicle crashes [1,2,3,4]. Since the introduction of rearward-facing CRS models, the design has evolved to further maximize the protection afforded. Some European rearward-facing CRS models are designed with a support leg (also referred to as a ‘load leg’) to reduce forward rotation during frontal impacts; this feature has been evaluated in laboratory-based sled tests. In an early study, Le Claire et al. [5] conducted sled tests using the R44 test bench and a vehicle seat to compare rearward-facing CRS models to the same CRS models that were modified to have a support leg. It was found that head acceleration values of anthropomorphic test device (ATD) in the modified rearward-facing CRS models with support legs were reduced compared to the unmodified CRS models for the R44 test bench, but not the vehicle seat. Sherwood et al. [6] compared results for the 12-month-old Child Restraint/Air Bag Interaction (CRABI-12) ATD in a rearward-facing CRS with a support leg to results for other rearward-facing CRS models in frontal impact sled tests with an impact speed of 49 km/h and a similar pulse shape to FMVSS 213 [7]. The rearward-facing CRS with a support leg had the lowest 15 ms Head Injury Criterion (HIC_15_) value and peak neck tensile force. In a subsequent study, Sherwood et al. [8] sled tested the CRABI-12 and Q1.5 ATDs in rearward-facing CRS models installed in a second-row minivan seat using a 47.5 km/h frontal crash pulse similar to FMVSS 213 [7]. Two rearward-facing European CRS models were tested, each with rigid lower anchors and a support leg, and compared to two US CRS models, each with flexible anchors and no support legs. Average HIC_15_ values for the European models were below injury tolerance values and significantly lower than values from the US models. In addition, peak neck tensile forces and neck flexion moments were typically lower for the rearward-facing European CRS models compared to the US models.

A recent study by Patton et al. [9] performed sled tests, which simulated the Consumer Reports CRS test method [10,11], to evaluate the effects of using a support leg in rearward-facing infant CRS models during frontal impacts. The Consumer Reports simulated test buck comprised a test bench seat and blocker plate with the latter representing a front row passenger seatback. The Consumer Reports simulated frontal impact crash pulse was based on full-scale frontal crashes into a rigid-barrier [12], which had a delta-v of 56 km/h and a peak acceleration of 35 g with an associated rise time of 35 ms. Patton et al. [9] found that the presence of a support leg in rearward-facing infant CRS models in frontal impacts was associated with reductions in head injury metrics for the CRABI-12 and Q1.5 ATDs. The presence of a support leg was also associated with increases in neck injury metrics for some combinations of CRS and ATD; however, neck injury metrics were below injury tolerance values for all tests with a support leg. For some tests that used the support leg, the CRS contacted the blocker plate, but the head of the ATD did not. In contrast, the CRS contacted the blocker plate for all tests that did not use a support leg and in some instances the head of the ATD also contacted the blocker plate.

Previous studies of support legs in rearward-facing infant CRS models have focused on frontal impacts; however, oblique principal directions of force have been found to be common in real-world crashes [13,14]. In a pure frontal direction, the load sustaining ability of the support leg is likely maximized. However, no study has investigated the effectiveness of support legs in a rearward-facing infant CRS for frontal-oblique impacts where the loading conditions likely reduce the forces sustained by the support leg compared to pure frontal crash conditions. Therefore, the aim of the current study was to evaluate the effectiveness of support legs in rearward-facing infant CRS models for frontal-oblique impacts. The secondary aim was to compare injury metrics with and without a blocker plate.

## 2. Materials and Methods

**Figure 1:**
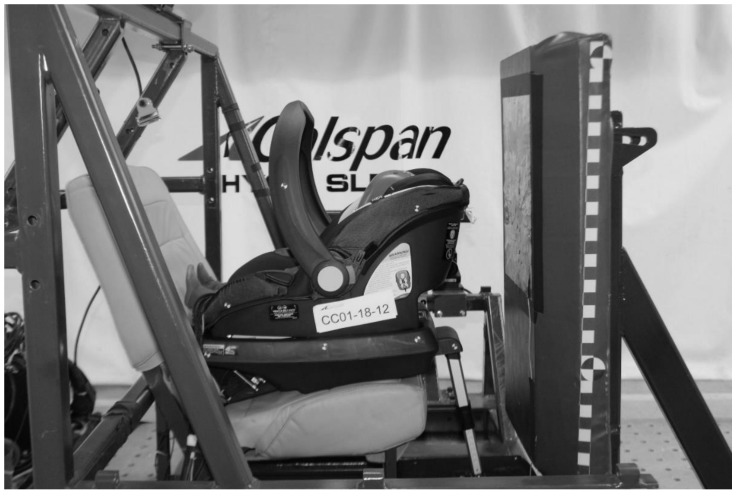
Child ATD seated in rearward-facing infant CRS with support leg installed in the simulated Consumer Reports test buck.

Sled tests were conducted using the simulated Consumer Reports test method as per Patton et al. [9] The test sled used in the current study is driven by a hydraulic-controlled gas-energized (HYGE) Crash Simulation System. The test buck comprised a test bench seat, blocker plate and sled-mounted high-speed camera system (Figure 1). The test bench seat was based on the second-row outboard seat of a 2010–2011 Ford Flex sport utility vehicle, which was rigidized to increase durability for repeated testing. The seatbelt was fixed at the shoulder belt D-ring and the outboard lap belt anchor. CRS and ATD installations were as specified in FMVSS 213 [7]. A force plate covered with non-slip grit tape was installed on the floor of the test buck to measure the ground reaction force during tests involving a support leg. The test buck faced the HYGE piston for frontal impacts and was rotated 30° for frontal-oblique impacts.

The blocker plate was used to represent the seatback of a front row seat and had the representative stiffness and geometry of front row passenger seatbacks from contemporary vehicles. The blocker plate comprised a rigid frame covered with a block of open-cell viscoelastic polyurethane foam (dimensions, 76 mm × 406 mm × 914 mm; density, 80 kg/m^3^), marine-grade vinyl upholstery and a conductive foil. Four high-speed video cameras were attached to an on-board camera frame, which provided top, rear and side views that moved with the test buck during each test. An additional high-speed video camera was mounted to the ceiling and provided a stationary top view of each test. The high-speed video cameras recorded frames with a resolution of 1600 × 1228 at a rate of 2000 s^−1^ for a duration of approximately 450 ms.

**Figure 2:**
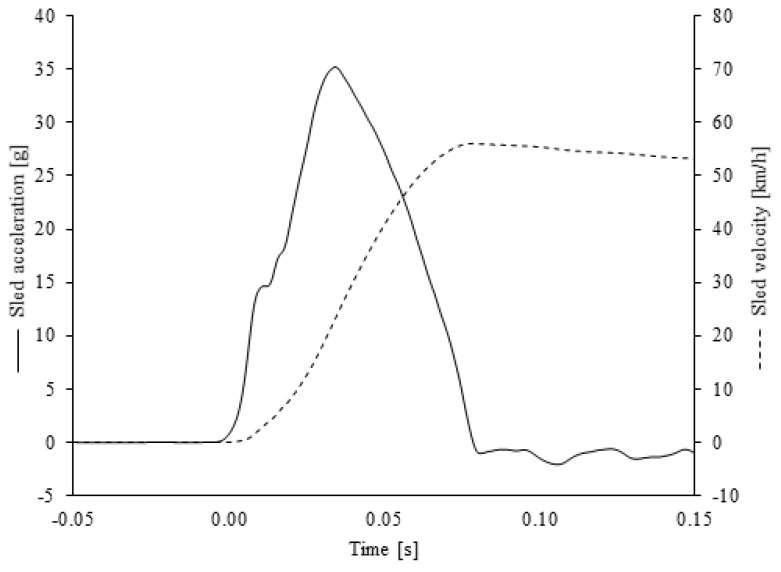
Exemplar sled acceleration (solid line) and velocity (dashed line) profiles for the simulated Consumer Reports frontal impact crash pulse.

The simulated Consumer Reports frontal impact pulse had a delta-v of 56 km/h, similar to the frontal impact using in the United States New Car Assessment Program (NCAP), and a peak acceleration of 35 g with a rise time to peak of 35 ms (Figure 2). The same frontal impact pulse was used for both the frontal and frontal-oblique tests.

Two exemplar rearward-facing infant CRS models, each with a support leg, were selected for the current study (Table 1): a rigid anchor infant child restraint system (RAICRS) and a flexible anchor infant child restraint system (FAICRS), which had two separate flexible connectors. When used, the support leg was positioned as specified by the manufacturer of the respective CRS. When the support leg was not used, it remained stowed in the available space underneath the CRS. A new CRS was used for each test.

A calibrated Q1.5 ATD was used to represent an infant aged 18 months: mass, 11.1 kg; height, 0.8 m [15]. The head of the ATD was instrumented with a 3-axis accelerometer located at the center of gravity of the head. The upper neck of the ATD was instrumented with a 6-axis load cell. A strip of conductive foil was attached to the top of the head of the ATD so that a voltage signal identified contact with the conductive foil on the blocker plate, herein referred to as head contact. Sled tests were conducted according to the test matrix (Table 2). Data from ATD instrumentation were filtered, as specified by SAE International J211 [16], and full time-histories of head accelerations and neck forces were extracted from which the following injury metrics were calculated: resultant head acceleration clip (3 ms), a_3_; head injury criterion (15 ms), HIC_15_; peak neck tensile force, F_Z_; and peak neck flexion moment, M_Y_.

Head and neck injury metrics for each condition were compared to previously published injury tolerance values (Table 3). Four multiple linear regressions, one for each injury metric (i.e., resultant head acceleration 3 ms clip, HIC_15_, peak neck tensile force and peak neck flexion moment), were used to identify significant (*p* < 0.05) associations between the test conditions (independent variables) and injury metrics (dependent variables). Test conditions (i.e., anchor type, support leg use, impact direction and blocker plate presence) were coded as categorical variables and two-way interactions of test conditions were also assessed. Linearity for the categorical independent variables was assumed and standard multiple linear regression diagnostics were performed for the normality (i.e., normal probability plot), homoscedasticity (i.e., scatterplots of standardized residuals) and multicollinearity (i.e., variance inflation factors) assumptions.

## 3. Results

**Figure 3:**
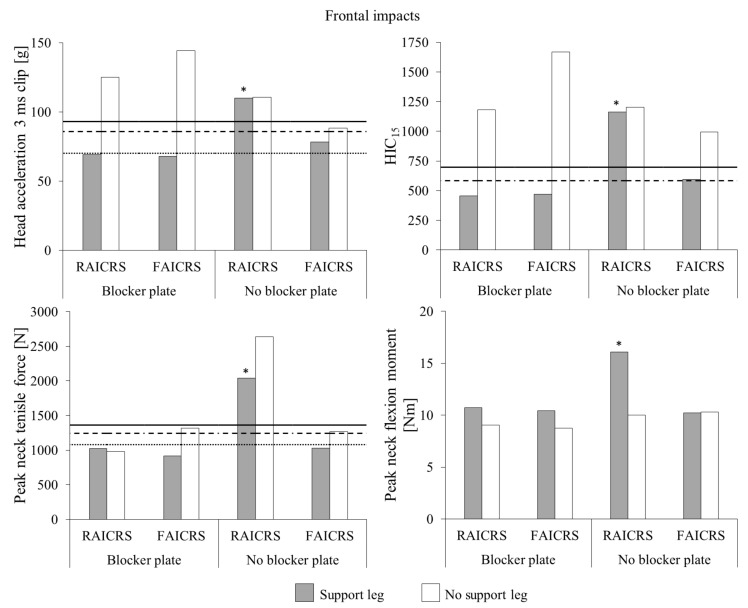
Head and neck injury metrics for the Q1.5 ATD in rearward-facing CRS models during frontal impacts with (shaded bars) and without (unshaded bars) a support leg. Q1.5 ATD head injury tolerance values (dotted lines) scaled from UN ECE R94 and 20% (dashed lines) and 50% (solid lines) risk of AIS3+ head injury tolerance values from Wismans et al. [17]. Note that there is no tolerance value scaled from UN ECE R94 for HIC_15_ and injury tolerance values for peak neck flexion moment are beyond the scale of the axis. Impact data for tests with the blocker plate were previously reported by Patton et al. [9]. RAICRS: rigid anchor infant child restraint system. FAICRS: flexible anchor infant child restraint system. * Denotes the impact test in which the support leg partially collapsed during the impact.

For frontal impact tests without the blocker plate (Figure 3), the head acceleration 3 ms clip value of the Q1.5 ATD in the flexible anchor infant CRS without the support leg exceeded the 20% risk of AIS3+ head injury tolerance value. The support leg was associated with a reduction of 11% head acceleration 3 ms clip, which fell below the 20% risk of AIS3+ head injury tolerance value. HIC_15_ of the Q1.5 ATD in the flexible anchor infant CRS without the support leg also exceeded the 50% risk of AIS3+ head injury tolerance value, but was reduced by 40% when the support leg was used to 596, which only slightly exceeded the 20% risk of AIS3+ head injury tolerance value of 585. For the flexible anchor infant CRS without the support leg, peak neck tensile force of the Q1.5 ATD slightly exceeded the 20% risk of AIS3+ neck injury tolerance value, which was reduced by 19% to below the neck injury tolerance value scaled from UN ECE R94 when a support leg was used. Although the presence of a support leg was associated with a negligible decrease and increase in peak neck flexion moments for the Q1.5 ATD in the flexible- and rigid-anchor infant CRS, respectively, all values were well below neck injury tolerance limits.

During the frontal impact test without the blocker plate, the support leg of the rigid anchor infant CRS partially collapsed. As a result, the head injury metrics for this test were similar to those for the no support leg test and exceeded the 50% risk of AIS3+ head injury tolerance values. Although a reduction of 23% was observed for peak neck tensile force of the Q1.5 ATD in the rigid anchor infant CRS when the support leg partially collapsed compared to the no support leg test, both values exceeded the 50% risk of AIS3+ neck injury tolerance value. The peak neck flexion moment of the Q1.5 ATD in the rigid anchor infant CRS when the support leg partially collapsed was elevated compared to the to the no support leg test, but both values were well below the injury tolerance values. The data from this test were removed from further analysis.

The frontal impact tests with the blocker plate were described by Patton et al. [9]. In short, the presence of a support leg for the rigid and flexible anchor rearward-facing CRS models was associated with reductions in Q1.5 ATD head injury metrics and peak neck tensile force to below injury tolerance values. Although peak neck flexion moments of the Q1.5 ATD in both rearward-facing CRS models with a support leg were elevated compared to the tests without a support leg, all values were well below injury tolerance values.

**Figure 4:**
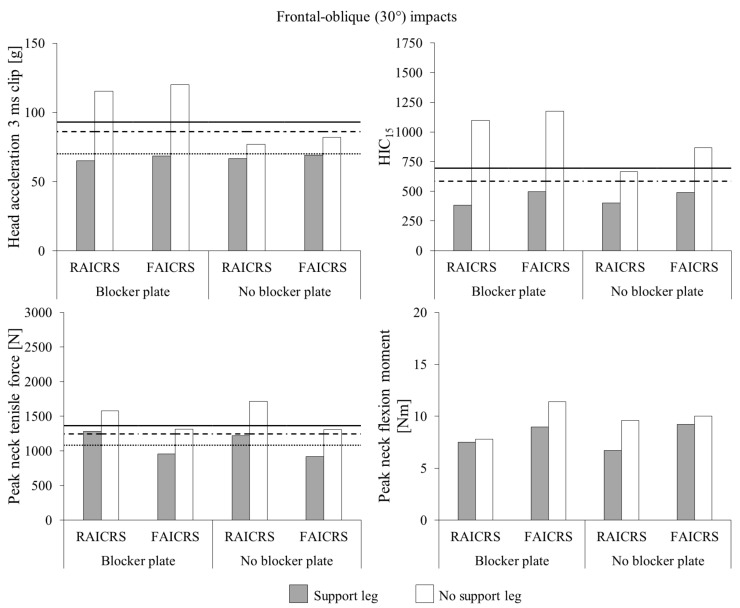
Head and neck injury metrics for the Q1.5 ATD in rearward-facing CRS models during frontal-oblique (30°) impacts with (shaded bars) and without (unshaded bars) a support leg. Q1.5 ATD injury tolerance values (dotted lines) scaled from UN ECE R94 and 20% (dashed lines) and 50% (solid lines) risk of AIS3+ injury tolerance values from Wismans et al. [17]. Note that there is no tolerance value scaled from UN ECE R94 for HIC_15_ and injury tolerance values for peak neck flexion moment are beyond the scale of the axis. RAICRS: rigid anchor infant child restraint system. FAICRS: flexible anchor infant child restraint system.

For frontal-oblique impact tests with and without the blocker plate (Figure 4), the presence of a support leg was associated with reductions in head acceleration 3 ms clip values of the Q1.5 ATD to below the injury tolerance value scaled from UN ECE R94. Similarly for HIC_15_ of the Q1.5 ATD, the presence of a support leg was associated with reductions in head acceleration 3 ms clip values to below the 20% risk of AIS3+ head injury tolerance value. For peak neck tensile force of the Q1.5 ATD in the rigid anchor infant CRS, reductions of 19% and 29% were associated with the use of a support leg for the blocker plate and no blocker plate conditions, respectively. Such reduced values were similar to the 20% risk of AIS3+ neck injury tolerance value of 1244 N. The use of a support leg for the flexible anchor infant CRS was associated with reductions of 27% and 30% for the blocker plate and no blocker plate conditions, respectively, which were both below the neck injury tolerance value scaled from UN ECE R94. Reductions in peak neck flexion moment were associated with the use of a support leg across CRS models and block plate conditions; however, all values were well below injury tolerance values.

When the blocker plate was used, no ATD head contact with the blocker plate was observed in all frontal and frontal-oblique impact tests with a support leg regardless of CRS model. In contrast, head contact with the blocker plate was observed for all frontal and frontal-oblique impact tests without a support leg.

The largest mean ground reaction force was 6231 N for the rigid anchor infant CRS support leg in frontal impacts (Table 4). Mean support leg ground reaction forces for the flexible anchor infant CRS were 27% and 31% lower than those from the rigid anchor infant CRS for frontal and frontal-oblique impacts, respectively. Mean support leg ground reaction forces from the rigid and flexible infant CRS models for the frontal-oblique impacts were 24% and 27%, respectively, lower than those for the frontal impacts.

The multiple linear regression models for independent variables of head acceleration 3 ms clip (*p* < 0.001), HIC_15_ (*p* < 0.001), peak neck tensile force (*p* = 0.004) and peak neck flexion moment (<0.001) were significant (Table 5) and all models satisfied the assumptions of linearity, normality, homoscedasticity and multicollinearity. The rigid anchor infant CRS was associated with significantly (*p* = 0.013) greater peak neck tensile force compared to the flexible anchor infant CRS. Use of a support leg was found to significantly reduce head acceleration 3 ms clip (*p* = 0.019), HIC_15_ (*p* = 0.0) and peak neck tensile force (*p* = 0.048). The frontal-oblique condition was found to significantly reduce head injury metrics (*p* < 0.01). In contrast, the presence of the blocker plate was found to significantly increase head acceleration 3 ms clip (*p* < 0.001) and HIC_15_ (*p* = 0.004). The rigid anchor infant CRS in the present of the blocker plate significantly reduced peak neck tensile force (*p* = 0.012). The use of a support leg in the presence of the blocker plate significantly reduced head acceleration 3 ms clip (*p* < 0.001) and HIC_15_ (*p* = 0.006). For frontal-oblique impacts, peak neck flexion moment was significantly increased (*p* = 0.016) in the presence of the blocker plate.

## 4. Discussion

Previous studies of support legs in rearward-facing infant CRS models have focused on frontal impacts and have found that the presence of a support leg is associated with a reduction in head injury metrics. However, real-world crashes often involve an oblique principal direction of force, but the effectiveness of support legs in rearward-facing infant CRS models have yet to be assessed for oblique impacts. Therefore, the current study investigated the effectiveness of support legs in rearward-facing infant CRS models for frontal-oblique impacts. In addition, a previous study used the blocker plate to represent the front row seatback; however, the influence of the blocker plate on ATD injury metrics in a rearward-facing infant CRS remains unknown. Therefore, the current study compared injury metrics for frontal and frontal-oblique impacts with and without a blocker plate for rearward-facing infant CRS with and without a support leg.

The presence of a support leg in both rearward-facing CRS models was associated with significant reductions of head injury metrics in the Q1.5 ATD, typically below injury tolerance limits, compared to the tests with no support leg. Such a finding supports the results of previous studies: that head injury metrics of child ATDs are reduced during frontal impacts when a support leg is used by the CRS [6,8,9]. The benefit of the support leg was particularly pronounced for tests with the blocker plate, with all head injury metrics being below injury tolerance limits. However, the fidelity of the interaction between the rearward-facing infant CRS and blocker plate as an adequate representation of the interaction that would occur in a real vehicle is not well understood.

The presence of the blocker plate was significantly associated with increased head injury metrics. In a previous study, Sherwood et al. [6] performed frontal impact sled tests the CRABI-12 ATD in three rearward-facing infant CRS models under different installation conditions for 49 km/h frontal impacts using the FMVSS 213 crash pulse. Two of the installation conditions were no structure in front and a rigid structure with a 150 mm gap. The rigid structure with a 150 mm gap condition had an increase of 31–169% for head acceleration 3 ms clip values and 199–275% for HIC_36_ values compared to the no structure in front condition. While the blocker plate used in the current study has a deformable surface with the representative stiffness and geometry of front row passenger seatbacks from contemporary vehicles, it has a rigid frame. However, there was a gap between the rear shell of the CRS and the blocker plate due to the positioning of the blocker plate for the simulated Consumer Reports test method. When the blocker plate was used, no ATD head contact with the blocker plate was observed when a support leg was used. In contrast, ATD head contact with the blocker plate was observed for all frontal and frontal-oblique impact tests without a support leg. Therefore, it is likely that the relatively greater head injury metrics for tests with the blocker plate, compared to tests without the blocker plate, were a result of the rear of the CRS impacting the blocker plate and/or, for tests without a support leg, the head of the ATD impacting the blocker plate.

Head injury metrics were significantly lower for frontal-oblique impacts compared to frontal impacts, which suggested that the latter is the worst-case scenario. However, oblique principal directions of force have been found to be common in real-world crashes [13,14]. Therefore, finding that head injury metrics were significantly reduced below injury tolerance levels for frontal-oblique impacts is an important step in understanding how the presence of a support leg affects head injury risk during a real-world crash.

For both infant CRS models, the presence of a support leg was associated with significant reductions of peak neck tensile force. Similarly, the presence of the blocker plate was associated with significant reductions of peak neck tensile force for the Q1.5 ATD in the rigid anchor infant CRS. Both the support leg and the blocker plate reduce peak neck tensile force by reducing the forward movement and rotation of the rearward-facing CRS during a frontal impact. Interestingly, the rearward-facing CRS with rigid anchors was significantly associated with increased peak neck tensile force compared to the CRS with flexible anchors. Charlton et al. [18] compared the performance of rearward-facing infant CRS models with rigid and flexible lower anchors in sled tests. For frontal impacts, maximum head excursion of the impact phase and HIC_36_ of the ATD were similar across anchor type; however, maximum head excursion of the rebound phase was greater for the ATD in the CRS with flexible anchors. It should be noted that the rearward-facing CRS models tested by Charlton et al. [18] had a top tether (also referred to as an ‘Australian tether’), which tethers the top of a rearward-facing CRS to a point in the vehicle aft of the CRS. A top tether performs the same function as a support leg: reducing forward rotation of a rearward-facing CRS [6]. In addition to the different anchor systems of the two CRS models tested in the current study, other design differences may be responsible for the significant difference in peak neck tensile forces.

The potential for support legs to damage floor pans has been anecdotally reported; however, the ground reaction forces for the support leg during frontal tests do not appear to be large enough to damage a floor pan. There are also concerns for support legs resting on footwell storage compartments [19,20]. Some European manufacturers require a foam filler to be fitted inside the storage compartment or for the storage compartment to be open and the support leg to extend to the base of the compartment [21]. In one frontal test, the support leg of the rigid anchor infant CRS partially collapsed, which resulted in similar head injury metrics to the frontal test without the support leg. Interestingly, Patton et al. [9] previously reported that the support leg of the flexible anchor infant CRS collapsed during a frontal impact. Although the simulated Consumer Reports frontal impact pulse is relatively severe, rearward-facing infant CRS support legs need to maintain integrity to effectively reduce rotation of the CRS and head injury metrics.

The main limitation of the current study was that the sled tests involved an idealized set of crash conditions for a simplified vehicle surrogate. For example, the material properties of the blocker plate had the representative stiffness of front row passenger seatbacks, but the seatbacks of some production vehicles may demonstrate better energy management than the blocker plate. In addition, the blocker plate has a rigid frame; however, vehicle seatbacks deform during frontal impacts [22]. Another limitation was that only single tests (i.e., no repeats) were performed for some conditions (i.e., frontal and frontal-oblique impact tests with no blocker plate). However, these tests are likely to have good repeatability, but the actual repeatability cannot be calculated without a substantial number of repeat tests for each condition. The two rearward-facing infant CRS models were selected for their features (i.e., support leg, flexible or rigid lower anchors) and may not represent the full range of CRS models available; other CRS with different design features may show varying results. For example, the CRS models with the support leg stowed in the current study may perform differently to CRS models that are not designed with a support leg. Although a frontal-oblique impact condition was tested, which is more representative of real-world crashes compared to a purely frontal crash, crash databases report a wide range of principal directions of force [13,14]. In addition, the simulated Consumer Reports frontal impact pulse used in the current study was relatively severe and likely rare in real-world collisions [9]. Future investigations of rearward-facing CRS models should perform sled tests with lower severity frontal impact pulses that are more representative of common real-world crashes.

## 5. Conclusions

In summary, the current study used sled tests to evaluate the effectiveness of support legs in rearward-facing infant CRS models for frontal and frontal-oblique impacts with and without a blocker plate. The presence of a support leg was associated with significant reductions of head injury metrics, typically below injury tolerance limits, particularly in the presence of the blocker plate. The presence of a support leg was also associated with significant reductions of peak neck tensile force. The presence of the blocker plate resulted in greater head injury metrics compared to tests without the blocker plate, but the result was non-significant. However, the fidelity of the interaction between the CRS and blocker plate as an adequate representation of the interaction that would occur in a real vehicle is not well understood.

## Figures and Tables

**Table 1:** Child restraint system models.

Model	Fixation	Facing	Restraint	Occupant Mass Range [kg]	Occupant Stature Limit [m]	Support Leg	CRS Mass [kg]
RAICRS	Rigid anchors	Rearward	5-point harness	1.8–14.5	0.813	Yes	10.4
FAICRS	Flexible anchors	Rearward	5-point harness	1.8–15.9	0.813	Yes	9.1

CRS: child restraint system. RAICRS: rigid anchor infant CRS. FAICRS: flexible anchor infant CRS.

**Table 2:** Text matrix.

ATD	Pulse	Direction	Blocker Plate	CRS Model	Support Leg	Number of Tests
Q1.5	Simulated Consumer Reports	Frontal	Yes *	FAICRS	Yes	2
					No	3
				RAICRS	Yes	3
					No	2
			No	FAICRS	Yes	1
					No	1
				RAICRS	Yes	1
					No	1
		Frontal-oblique (30°)	Yes	FAICRS	Yes	2
					No	2
				RAICRS	Yes	2
					No	2
			No	FAICRS	Yes	1
					No	1
				RAICRS	Yes	1
					No	1

ATD: anthropomorphic test device. CRS: child restraint system. RAICRS: rigid anchor infant CRS. FAICRS: flexible anchor infant CRS. * Impact data for frontal tests with the blocker plate were previously reported by Patton et al. [9].

**Table 3:** Head and neck injury tolerance values for the Q1.5 ATD.

Source	Method	a_3_ [g]	HIC_15_	+F_Z_ [N]	+M_Y_ [Nm]
Wismans et al. [17]	Scaled data from UN ECE R94	70		1080	48
	20% risk AIS3+ (certainty method)	79	585	1244	61
	50% risk AIS3+ (certainty method)	86	696	1364	74

**Table 4:** Support leg ground reaction forces.

Impact	CRS	Support Leg Ground Reaction Force [N]	
		Mean	SD
Frontal	RAICRS	6231	438
	FAICRS	4522	53
Frontal-oblique (30°)	RAICRS	4741	98
	FAICRS	3282	44

CRS: child restraint system. SD: standard deviation. RAICRS: rigid anchor infant CRS. FAICRS: flexible anchor infant CRS.

**Table 5:** Multiple linear regression of head and neck injury metrics.

	Head Injury Metrics						Neck Injury Metrics					
	a_3_ [*g*]			HIC_15_			F_Z_ [N]			M_Y_ [Nm]		
	Coeff.	SE	*p*-Value	Coeff.	SE	*p*-Value	Coeff.	SE	*p*-Value	Coeff.	SE	*p*-Value
Intercept	99.4	7.3	<0.001	1169	109	<0.001	1592	183	<0.001	9.80	0.51	<0.001
Rigid anchors	3.3	9.2	0.727	-107	137	0.446	655	229	0.013	0.51	0.64	0.440
Support leg	−24.3	9.2	0.019	−607	137	<0.001	−497	229	0.048	0.59	0.64	0.371
Frontal-oblique	−22.2	8.5	0.020	−381	126	0.009	−397	211	0.082	1.12	0.59	0.079
Blocker plate	40.1	7.8	<0.001	405	117	0.004	−401	196	0.060	−0.86	0.55	0.138
Rigid anchors × support leg	5.8	7.7	0.468	150	116	0.216	82	193	0.678	0.58	0.54	0.303
Rigid anchors × Frontal-oblique	−2.1	7.7	0.788	29	115	0.805	46	193	0.817	−2.56	0.54	<0.001
Rigid anchors × blocker plate	−12.2	8.9	0.193	−161	133	0.245	−643	222	0.012	−0.61	0.62	0.342
Support leg × frontal-oblique	13.1	7.7	0.113	247	115	0.050	−53	193	0.787	−2.92	0.54	<0.001
Support leg × blocker plate	−44.1	8.9	<0.001	−429	133	0.006	218	222	0.343	0.76	0.62	0.240
Frontal-oblique × blocker plate	6.6	8.9	0.465	72	132	0.595	606	221	0.016	0.82	0.62	0.205
R^2^ (adjusted)	90.0%			90.0%			60.9%			75.4%		
F	20.70 (<0.001)			22.67 (<0.001)			4.74 (0.004)			8.36 (<0.001)		

a_3_: head acceleration clip (3 ms). HIC_15_: head injury criterion (15 ms). F_Z_: peak neck tensile force. M_Y_: peak neck flexion moment. Coeff.: coefficient. SE: standard error.

## Data Availability

Data available upon reasonable request.
